# Microglia-Induced Maladaptive Plasticity Can Be Modulated by Neuropeptides *In Vivo*

**DOI:** 10.1155/2015/135342

**Published:** 2015-07-26

**Authors:** Stefano Morara, Anna Maria Colangelo, Luciano Provini

**Affiliations:** ^1^Neuroscience Institute (CNR), Via Vanvitelli 32, 20129 Milano, Italy; ^2^Department of BIOMETRA, University of Milano, Via Vanvitelli 32, 20129 Milano, Italy; ^3^Laboratory of Neuroscience “R. Levi-Montalcini”, Department of Biotechnology and Biosciences, University of Milano-Bicocca, Piazza della Scienza 2, 20126 Milano, Italy; ^4^SYSBIO Centre of Systems Biology, University of Milano-Bicocca, Piazza della Scienza 2, 20126 Milano, Italy; ^5^NeuroMI Milan Center for Neuroscience, University of Milano-Bicocca, 20126 Milano, Italy

## Abstract

Microglia-induced maladaptive plasticity is being recognized as a major cause of deleterious self-sustaining pathological processes that occur in neurodegenerative and neuroinflammatory diseases. Microglia, the primary homeostatic guardian of the central nervous system, exert critical functions both during development, in neural circuit reshaping, and during adult life, in the brain physiological and pathological surveillance. This delicate critical role can be disrupted by neural, but also peripheral, noxious stimuli that can prime microglia to become overreactive to a second noxious stimulus or worsen underlying pathological processes. Among regulators of microglia, neuropeptides can play a major role. Their receptors are widely expressed in microglial cells and neuropeptide challenge can potently influence microglial activity *in vitro*. More relevantly, this regulator activity has been assessed also *in vivo*, in experimental models of brain diseases. Neuropeptide action in the central nervous system has been associated with beneficial effects in neurodegenerative and neuroinflammatory pathological experimental models. This review describes some of the mechanisms of the microglia maladaptive plasticity *in vivo* and how neuropeptide activity can represent a useful therapeutical target in a variety of human brain pathologies.

## 1. Introduction

Inflammation developed as a defensive beneficial process that can protect from an external insult. In a simplistic manner, following infection or trauma a defensive barrier is created by the activation of specific cellular and molecular inflammatory mechanisms that kill pathogens or infected cells and eliminate dying/dead cells and clear debris while secreting cytokines and chemokines (among other factors) to orchestrate a multicellular response. This process is accomplished, in the periphery, by the array of immune cells that sense the danger, migrate, and recruit additional defenders to attack the intruders or protect tissues from damaged cells. When the defensive/reparative effect is achieved, the inflammatory response is dampened and finally resolved [1, 2]. Such homeostatic process requires both (i) a continuous surveillance of the environment performed by specialized cells that monitor changes and counteract any danger and (ii) the capacity to stop the defensive action (to resolve inflammation).

Chronic inflammation, instead, occurs when the resolving process fails and the defensive inflammatory cells trigger a self-sustained process that continues in the absence of underlying dangers, creating a new one. This constitutes what is now referred to as a maladaptive process and leads to a broken homeostatic equilibrium. To provide a few examples, this process is thought to occur in pulmonary hypertension [3], ischemic acute kidney injury [4], and coronary artery disease induced by atherosclerosis [5]. A classic example of maladaptive response is allergic asthma that can occur following repeated exposure to allergenic or viral agents during infancy [6]. Allergic asthma is thought to be driven by altered responses of Th2 and Th17 lymphocytes (but also type-2 innate lymphoid cells) with the intervention of specific molecular pathways involving complement cascade components and a fibrinogen/proteinases/toll-like receptor (TLR) sequence [7–10].

It is worth mentioning that among immune cells macrophages (the peripheral counterparts of microglia) have been claimed to be involved in maladaptive responses. For example, cardiac interstitial fibrosis is exacerbated after myocardial infarction by caveolin-1 deletion that promotes an unbalanced M2 macrophage activation [11], whereas altered lipid metabolism and accumulation of cholesterol-laden macrophages in the artery wall cause a chronic inflammation leading to atherosclerosis [12].

## 2. Brain: Homeostasis and Maladaptive Responses

Homeostasis is needed within the central nervous system (CNS) also. The specificity of the structures and mechanisms that sustain CNS functioning requires a tightly controlled, delicate balance in and around neuronal and glial cells. The rapid and continuous exchanges of patrolling immune cells must be avoided in the brain under physiological conditions (or at least reduced at a much lower level than the one attained in the periphery): such avoidance has been accomplished through what is referred to as “immune-privilege/specialization” of the brain, which implies, for example, the relative inaccessibility to the brain parenchyma by the peripheral immune cells (see, however, the detailed discussions in [13, 14]). The brain homeostasis is thus tightly regulated by specific resident effectors, the main one being microglia.

Microglia are emerging as crucial players in brain functioning (and development) [15] and the number of papers reporting on them has increased exponentially during the last few years. They are the primary immune cells in the CNS and have been historically classified in morphological and functional terms as subdivided in three different forms, the ramified-resting, the bushy-activated, and the ameboid, macrophage-like, phagocyting form [15]. A major breakthrough about their primarily immunological/inflammatory role came from time-lapse two-photon imaging experiments that showed that the fine processes and protrusions of ramified/resting microglial cells are extremely motile in the intact mouse cortex and continually survey their microenvironment [16, 17]. Following focal traumatic brain injury the processes rapidly converge on the site of injury (without cell body movements) and shield the healthy tissue from the injured one [16, 17]. Subsequently, the same technical approach allowed discovering that fine microglial processes make direct contacts with neuronal synapses that in the intact brain are retracted within a time scale of a few minutes, whereas, following transient cerebral ischemia, they are kept for much longer time (about one hour) and are frequently followed by the disappearance of the presynaptic terminal [18]. Soon after that, another major breakthrough was the demonstration that the physiologically occurring synaptic pruning during postnatal development requires the active involvement of microglia that phagocyte synaptic material: this process is mediated by the fractalkine receptor Cx3cr1 and plays a major role in normal brain developmental wiring [19]. Very interestingly, the lack of Cx3cr1 caused a transient reduction of microglia during a critical postnatal period with a consequent deficit in synaptic pruning, associated with brain functional and connectivity alterations, deficits in social interaction, and increased repetitive-behavior (phenotypes that are considered typical of autism and other neurodevelopmental and neuropsychiatric disorders) [20]. The developmental synaptic pruning, as it occurs during the peak of postnatal retinogeniculate system in the mouse, was shown to be executed by microglia engulfment of presynaptic inputs in a neural activity- and complement receptor 3/C3-dependent manner [21]. However, the interaction between neurons and microglia during the critical period of activity-dependent neural circuit refinement is bidirectional. Time-lapse imaging in the optic tectum of larval zebrafish has shown that locally elevated neuronal activity guides ramified (resting/surveying) microglial processes to contact highly active neurons (a process requiring active pannexin-1 hemichannels in neurons and small Rho GTPase Rac in microglia) and, reciprocally, microglia reduce both spontaneous and visually evoked activities of contacted neurons [22].

## 3. Microglia Maladaptive Responses

Thus, microglia functioning is regulated by the neural microenvironment and, in turn, microglia play critical roles in developmental circuit reshaping and adult brain physiological and pathological surveillance. In particular, a homeostasis alteration induces a cascade of conserved adaptive microglial responses involving biochemical, physiological, and morphological changes associated with the production of mediators that restore brain homeostasis. However, severe or prolonged alterations (such as those occurring in neurodegeneration, traumatic brain injury, stress, ageing, and neuroinflammation, but also chronic systemic inflammation) can have profound effects on microglia phenotype and functional activity which is now usually referred to as “priming” and currently the subject of intense analysis [23–27]. There is a substantial amount of clinical and experimental evidence indicating that the presence of a diseased state results in the priming for exaggerated responses to subsequent challenges or, on the contrary, a second injury aggravates the course of the first one. In experimental conditions this was described also in the case of microglial priming.* In vivo* primed microglia were described, for example, by using a prion disease model. In such a model, the first clinical signs appeared at week 23 after induction, but microglial activation was already detectable at week 8: systemic (but also intracerebral) LPS challenge at week 19 induced a greater production of proinflammatory mediators and a corresponding increased microglial expression of IL-1 in brain of diseased mice than in control brains [28]. Primed microglia have been proposed to be characterized by a profile consisting of (1) higher basal expression of inflammatory mediators and markers, (2) lower activation threshold, and (3) exaggerated inflammatory response, in comparison to unprimed microglia [25]. In the same prion model, a second experiment showed that a systemic challenge of polyinosinic:polycytidylic acid (a toll-like receptor-3 agonist mimicking inflammatory responses to systemic viral infection) accelerated underlying prion disease [29]. A similar situation was detected in a rat Parkinson model, obtained by 6-hydroxydopamine (6-OHDA) injection in the striatum, in which microglial activation is associated with neurodegeneration in the substantia nigra. In such a model, intracerebral injection of lipopolysaccharide (LPS) or chronic systemic IL-1 administration caused exacerbation of neurodegeneration and increased microglial activation in the substantia nigra [30]. In a second Parkinson model, two systemic injections of paraquat were used to induce a dopaminergic cell loss in the substantia nigra [31]. A single paraquat administration could only induce microglial activation whereas dopaminergic cell loss and oxidative stress in the substantia nigra could be elicited only after the second administration. However, if the first administration was subsequently blocked by minocycline (a microglia activation inhibitor), the second paraquat administration failed to induce any effect. On the other hand, systemic administration of LPS induced microglial activation in the substantia nigra two days after injection: at this time, a single paraquat administration was sufficient to induce nigral cell loss [31].

These experiments show that microglia priming is a critical step that leads to aggravate preexisting diseases and worsen the outcome of a secondary challenge. This was seen, for example, in traumatic brain injury in aged animals. Compared to young animals, aged ones show a higher inflammatory profile of microglia [32]. In a traumatic brain injury model (controlled cortical impact) aged animals developed larger lesions and increased neurodegeneration, associated with increased microglia activation, M1/M2 balance switch, and increased oxidative stress in comparison to young animals [33]. These effects could be the results of a switch in the activity profile of microglia induced by a first alteration in homeostasis towards an injury-dependent primed state and lead subsequently to altered responsiveness to a second challenge [34]. Indeed, stress might induce a priming stimulus for microglia via increased high mobility group box-1 protein and, in turn, produce microglial overreactivity to LPS challenge via the nucleotide-binding domain, leucine-rich repeat, and pyrin domain containing protein 3 inflammasome [35].

If there is a preexisting pathological condition the outcome of a secondary challenge could be worse than under unimpaired conditions or could aggravate the preexisting condition but also provoke unexpectedly different symptoms: for example, a secondary inflammatory challenge associated with preexisting traumatic brain injury could induce a depressive-like behavior (in addition to increased inflammatory cytokine production in microglial cells) which is not detected in uninjured animals [36]. This effect should not be unexpected, as the involvement of microglia in psychiatric, psychological, and cognitive diseases is more and more recognized [37, 38].

## 4. Mediators of Microglia Maladaptive Responses

The factors that may induce a primed state and hence a possible subsequent maladaptive response in microglia are several and they could lead to developing differential priming effects. Apart from physiological processes (such as aging) or injuries (such as trauma, both possibly involving oxidative stress), neurodegenerative processes, or prion disease inducers, specific factors have been identified and some have already been mentioned in the previous section: neurotoxins (6-OHDA, 1-methyl-4-phenyl-1,2,3,6-tetrahydropyridine, MPTP or paraquat) either systemically or directly injected into CNS parenchyma [31, 39, 40], high mobility group box-1 which mediate the inescapable shock stress-induced priming effect [35], TLR ligands [41, 42], amyloid-*β* (A*β*) [43], chronic ethanol exposure [44], alpha-synuclein [45], beta-adrenergic receptor activation [46], granulocyte macrophage-colony stimulating factor (GM-CSF) [47], mycobacteria-infected macrophages [48], and DNA repair deficiency [49]. As already mentioned, oxidative stress could also provide a priming mechanism, as it occurs during aging, following ischemia or neurotoxin exposure, and it is here described more in detail.

## 5. Microglia and Oxidative Stress

Although it is still not clear whether oxidative stress is the initiating event associated with neurodegeneration, several data indicate that it is common to all neurodegenerative disorders, including Alzheimer's disease, Parkinson's disease, multiple sclerosis, and amyotrophic lateral sclerosis. Oxidative stress, due to accumulation of reactive oxygen species (ROS) (that include superoxide (O_2_^−^), hydrogen peroxide (H_2_O_2_), and hydroxyl radical (OH^−^)) and nitric oxygen (RNS) species, is the result of an imbalance between generation of free radicals and their elimination by endogenous antioxidant mechanisms.

**Figure 1:**
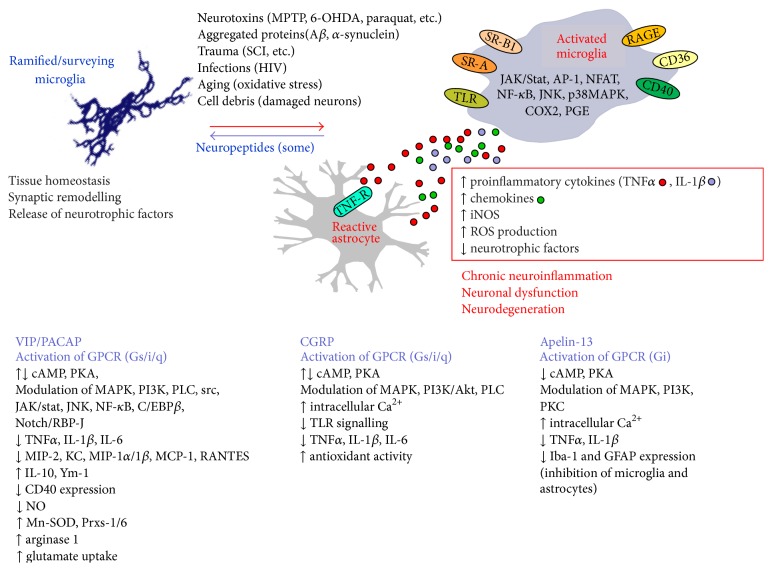
Schematic summary of microglia roles in physiological and pathological conditions in relation with the neuropeptide homeostatic influence on microglia. MPTP: 1-methyl-4-phenyl-1,2,3,6-tetrahydropyridine; 6-OHDA: 6-hydroxydopamine; A*β*: beta-amyloid; SCI: spinal cord injury.

Activated microglia (described also as reactive microgliosis) have been shown to be an important source of ROS in response to brain injury, ischemia, or inflammatory stimuli [50–52], through mechanisms leading to stimulation of NADPH oxidase and nitric oxide synthases (NOS) activity [53–56]. This function of microglia is linked to the expression of multiple pattern recognition receptors that identify a wide number of neurotoxic stimuli. Among them, distinct members of the TLR family, in particular TLR 1–9, are essential components of the microglial innate immune response [57, 58]. TLR ligands also engage scavenger receptors (SR) that recognize modified lipoproteins and polyanionic ligands. SR-A, SR-B1, CD36, and receptor for AGEs, advanced glycation endproducts (RAGE), in particular, participate in microglial activation and ROS production in response to A*β* fibrils [59–61] (Figure 1).

Microglial pattern recognition receptors also include integrin CD11b/CD18 (MAC1, macrophage antigen complex-1, also known as complement receptor 3, CR3) that mediates LPS-induced production of superoxide and whose expression has been found to be elevated in the brains of* post mortem* Alzheimer's disease patients [62, 63].

Interaction of pattern recognition receptors with specific ligands stimulates NADPH oxidase and iNOS leading to production of neurotoxic factors, including toxic amounts of superoxide (O_2_^−^) and nitric oxide (NO^∙^) free radicals, as well as increased levels of proinflammatory cytokines (IL-1*β*, TNF*α*, and IL-6). Intracellular ROS and NO act as second messengers through kinase cascades (MAPKs and PI3 kinase) and transcription factors (NF-*κ*B, Nrf2, and AP-1) that modulate both microglial proinflammatory function and survival [54, 55] (Figure 1). However, dysregulation of intracellular ROS in microglia amplifies proinflammatory gene expression. Moreover, NO^∙^ and O_2_^∙−^ rapidly react to form the strong oxidant peroxynitrite (ONOO^−^), thus contributing to the progressive nature of microglia-mediated neurotoxicity. ROS, mainly generated at complex I (NADPH ubiquinone oxidoreductase) or ubiquinone site of complex III (ubiquinone-cytochrome c reductase) of the mitochondrial transport chain, further impair mitochondrial electron transport and enhance ROS production.

Increased production of ROS and proinflammatory mediators, together with the decreased secretion of neurotrophic factors, triggers neuronal homeostasis. Oxidative stress leads to mitochondrial dysfunction: decreased mitochondrial membrane potential and opening of mitochondrial permeability transition pores lead to collapse of energy-dependent ion transport, ATP depletion, and intracellular Ca2^+^ overload. These biochemical changes also trigger excitotoxicity caused by inhibition of glutamate uptake by the astrocytic Na^+^-dependent glutamate transporter-1 (GLT-1, EAAT2) and glutamate-aspartate transporter (GLAST, EAAT1), most likely due to their degradation by Ca^2+^-activated (*μ*)-calpains [64, 65]. Increased extracellular glutamate is also determined by nonspecific release of L-Glu by activated microglia [66] and by alteration of the glutamate/cystine antiporter, which exchanges internal glutamate for cystine [67]. EAATs seem to be expressed by microglia only under pathological situations, like following infectious diseases [68]. The relevance of neuroinflammation was also proved by the inverse association of nonsteroidal anti-inflammatory drug use with the risk of Parkinson's disease in two prospective studies for nonsteroidal anti-inflammatory drug and aspirin [69]; moreover, minocycline treatment was found to reduce neuroinflammation following paraquat exposure [31].

Age-dependent alteration of gene expression and accumulation of ROS appear to be the most relevant factors for aging and age-related disorders. Normal aging is characterized by a mild chronic inflammatory activity and imbalance between proinflammatory (TNF*α*, IL-1*β*, and systemic IL-6) and anti-inflammatory cytokines (IL-10) in both the blood and the brain [70, 71]. These aging-associated changes are believed to be responsible for increased neuronal vulnerability to synaptic damage and degeneration [72].

Neurons are particularly susceptible to oxidative damage due to their dependence on oxidative phosphorylation for their large energy demand. Moreover, the brain is rich in fatty acids, including polyunsaturated fatty acids, which are more prone to peroxidation and may trigger a chain reaction of lipid peroxidation in biomembranes. Under physiological conditions, intracellular ROS and NO act as second messengers and activate survival signalling. The increase of free radicals causes oxidative damage to proteins, lipids, and DNA and leads to decreased mitochondrial membrane potential and ATP depletion. Accumulation of lipid hydroperoxides alters membrane permeability and fluidity and oxidizes membrane proteins, leading to alterations in ion transport and intracellular flux of Ca^2+^. Mitochondrial dysfunction can also determine the release of cytochrome c and activation of intrinsic mitochondrial apoptosis.

In microglia, as in all other cell types, redox homeostasis is maintained by cellular and extracellular redox buffering systems, including the redox couples GSH/GSSG (glutathione-glutathione disulfide), cysteine/cystine, oxidized/reduced thioredoxin, and key antioxidant enzymes, including superoxide dismutase, catalase, the selenoproteins glutathione peroxidase, and thioredoxin reductase [73], as well as nonenzymatic antioxidants such as *α*-tocopherol (vitamin E), ascorbate (vitamin C), *β*-carotene, and flavonoids. It is worth noting that a number of natural antioxidants and anti-inflammatory compounds have been shown to provide effective neuroprotection against oxidative stress, in both* in vitro* and several models of age-related neurodegenerative conditions linked to oxidative stress, such as those involved in Alzheimer's disease (A*β* and tau toxicity), Parkinson's disease (*α*-synuclein, 6-OHDA, and MPTP), multiple sclerosis (experimental autoimmune encephalopathy, EAE, model), and ischemia (oxygen-glucose-deprivation) [74–77]. Antioxidant dietary supplements include polyphenols (flavonoids and resveratrol), carotenoids (lycopene), thiolic compounds (such as *α*-lipoic acid, glutathione, and N-acetylcysteine), and oligoelements (such as selenium) which are thought to exert their action on the basis of their immunomodulatory and anti-inflammatory properties, as well as the capability to activate sirtuins, transcription factors (in particular, NF-*κ*B, Nrf2, and PPAR/PGC-1*α*), and pathways that regulate metabolism, antioxidant responses, and cellular homeostasis [78].

## 6. Microglia Maladaptive Response Regulators

At present, there is an active search for microglia maladaptive response blockers. Among other factors, neuropeptides are emerging as new players that influence microglia activation and thus can play such a role [79]. A large array of studies shows the specific modulatory activity of many neuropeptides on microglial cells* in vitro*; but, in order to better relate their activity with microglia maladaptive responses* in vivo*, the present review focuses on the* in vivo* interaction between neuropeptides and microglia. In particular, the present review analyzes* in vivo* examples that describe the association of beneficial effects of neuropeptides on brain disease model symptoms with the regulation of inflammatory microglia activation (Table 1).

We will focus on presumably direct effects of neuropeptides on microglia by “brain delivery” (direct acute or chronic administrations or production through viral vectors). Caution is required in attributing the amelioration to a “direct brain effect” even in the case of “brain delivery,” due to possible indirect effects through neuropeptide leakage into the bloodstream or activation of the tiny minority of nonresident immune cell that travel in and out CNS (see, e.g., [80]). However, chronic intrathecal delivery of CGRP by osmotic minipumps (in the lumbar CSF) did not produce significant changes in peripheral lymphocytes in a multiple sclerosis model, the (chronic) EAE [81].

A summary of (some) neuropeptide action and their relevance for microglia-induced maladaptive plasticity is depicted in Figure 1.

## 7. VIP/PACAP Family

Vasoactive intestinal peptide (VIP) and pituitary adenylate cyclase activating polypeptide (PACAP) are neuropeptides that belong to a family that includes peptide histidine-isoleucine (PHI), peptide histidine-methionine (PHM), secretin, glucagon, glucagon-like peptide (GLP), glucose-dependent insulinotropic polypeptide (GIP), growth hormone releasing hormone (GHRH), and helodermin [82]. VIP and PACAP signalling is cell type- and context-dependent and is mediated by the activation of the specific G protein-coupled receptors, VPAC1/VIPR1, VPAC2/VIPR2, and ADCYAP1R1/PAC1, which are coupled primarily to Gs that activates adenylate cyclase and in turn protein kinase A (PKA) [83], as well as exchange proteins activated by cAMP [84]. In parallel, additional pathways can be activated or inhibited in specific cells, including MAPK [85–87], phospholipase C [88], phosphatidylinositol 3-kinase [89], NO [90], src [91], Jak/STAT, and NF-*κ*B [92, 93].

VIP and PACAP immunological actions have been extensively analyzed* in vitro* and* in vivo* and are found to be frequently superimposable. VIP can affect innate and adaptive immunity and is thought to exert primarily anti-inflammatory roles [94, 95]. Many effects of VIP, such as those directed towards influencing T cell differentiation and function, seem to be exerted both directly and indirectly through the immunomodulation of dendritic cells. In such a context, VIP-differentiated dendritic cells were shown to be able to restore immune tolerance* in vivo*, to facilitate transplantation by reducing the deleterious consequences of acute graft-versus-host disease [96]. Long lasting VIP secretion by lentiviral-VIP transduced dendritic cells had a therapeutic effect on EAE and cecal ligation and puncture sepsis models [97].

In addition to dendritic cells, macrophages are also VIP targets. For example, the peptide can inhibit phagocytosis and chemotaxis of alveolar macrophages* in vitro* [98] and, together with PACAP, enhance macrophage resistance to HIV-1 infection by decreasing viral growth [99]. Also the brain counterpart of macrophages, microglia, can be modulated by VIP/PACAP* in vitro*. In particular, PACAP activates a potassium-outward current [100] and inhibits CD40 expression [101], whereas both (with different potency) are able to inhibit inflammatory cytokines (TNF*α*, IL-1*β*, and IL-6), chemokines (MIP-2, KC, MIP-1*α*, MIP-1*β*, MCP-1, and RANTES), and NO in LPS-activated microglia [102–104], inhibit concurrently MAKK1/MEK4/JNK, CBP-NF-*κ*B interaction, and stimulate JunB production [105, 106].

### 7.1. VIP

As far as CNS is concerned,* in vivo* experimental models of brain injuries or diseases were analyzed for VIP involvement [95]. An interesting discovery has been that VIP is upregulated in microglia following cerebrum cold injury and the VIP/VPAC2 system can induce reactive astrocytosis and neuroprotection against the injury-induced excitotoxicity [107]. Delgado and Ganea [108, 109] injected VIP in the brain to examine the effects of the peptide in two models, cerebral trauma and MPTP-induced dopaminergic cell loss (Parkinson's disease model). In both experimental paradigms a robust inflammatory microglia activation accompanies the pathogenesis, although its possible causative role is still a matter of investigation. In the brain trauma model (a stab wound operation), an intracerebroventricular injection of VIP, made at the same time of trauma induction, reduced neurodegeneration, recruitment of mononuclear phagocytes, inflammatory microglia activation, and local production of TNF*α* and IL-1*β* [108]. In Parkinson's disease model, an acute dosing of MPTP (20 mg/kg intraperitoneally injected every 2 h for four doses) was used to induce dopaminergic cell loss in the substantia nigra [109]. A single VIP injection into the substantia nigra was effective in reducing the loss of dopaminergic neurons in the substantia nigra itself and of dopaminergic fibers in the striatum. Interestingly, the neuroprotective effect was associated with reduced inflammatory microglia activation in the substantia nigra and striatum and with a local reduction of TNF*α* expression in both structures [109]. It should be noted that the neuroprotective action was effective only if VIP injection was made within a short time after the first MPTP injection (0.5–3 hours). A third example comes from a study on a model of Alzheimer's disease, APPswe/PSEN1de9 transgenic mice. In this model, intracerebroventricular injection of recombinant adenovirus vector encoding functional VIP led to accumulation and activation of CD11b-positive microglia and a PKC-dependent increase in microglial phagocytosis of fibrillar A*β*42, hence attenuating amyloid deposition [110].

In the context of primed inflammatory microglia, it might be worth mentioning that VIP can decrease the inflammation-induced increase in TLR expression [111] by a homeostatic mechanism [112, 113]. In the infected cornea VIP was able to downregulate proinflammatory TLRs and upregulate anti-inflammatory TLRs [114]. Moreover, VIP can reverse TLR4 signalling in rheumatoid arthritis synovial fibroblasts [115]. Finally, VIP can suppress oxidative stress in several cell types, both* in vitro* [116–118] and* in vivo* [119, 120].

### 7.2. PACAP

In addition to the described effects on microglia* in vitro* (see above), PACAP can protect from oxidative stress in nonneural [121, 122] but also glial cells [123]. But PACAP exerts a similar role* in vivo* also, as it was seen in knockout and wild-type animals [124], renal ischemia/reperfusion injury [125], and global cerebral ischemia [126].

The action of PACAP as regulator/reparative mediator acting on microglia cells* in vivo* was tested in a model of spinal cord injury. By using a weight-drop device [127] local injections of PACAP in synergy with human mesenchymal stem cells increased neurofilament (NF200) immunoreactivity and the levels of antioxidant enzymes, such as Mn-superoxide dismutase and peroxiredoxin-1/6, in the injured spinal cord, and led to better locomotor functional recovery [128].* In vitro*, PACAP applied to microglia cocultures with hMSCs increased a subpopulation of microglia expressing galectin-3 and the uptake of extracellular glutamate by astrocytes, suggesting that the* in vivo* action could be mediated possibly by microglia. In a mouse stroke model, PACAP-producing stem cells were transplanted intracerebroventricularly three days after permanent focal ischemia. These cells promoted a functional recovery associated with the modulation of key transcriptional factors, such as NF-*κ*B, C/EBP-*β*, and Notch/RBP-J, decreased expression of TNF*α*, increased expression of IL-10 and Ym-1, and increased number of arginase-1+ cells, suggesting a redirection of the microglial phenotype towards a neuroprotective M2 type [129].

The role of VIP and PACAP in this subject, however, should be analyzed taking into account the differential expression and specific involvement of their receptors. For example, in the hippocampus PAC1, mRNA expression is increased at 7 days following ischemia, VPAC1 mRNA is decreased at 3 days, and VPAC2 mRNA substantially unchanged [130] whereas in a model of reactive gliosis PAC1 and VPAC2 (but not VPAC1) mRNA are increased in astrocytes (and VIP in microglia) [107]. As to the role of specific VIP/PACAP receptors in microglia, it is worth mentioning that microglial VPAC1 mediates proliferative and trophic effects on neural stem/progenitor cells, a role which might have relevant implications in neuroinflammatory and neurodegenerative diseases [131].

## 8. CGRP

Calcitonin Gene-Related Peptide (CGRP) is a neuropeptide that belongs to a family that includes adrenomedullin, amylin/islet amyloid polypeptide (IAPP), and calcitonin, it is mainly expressed in the CNS, by neuronal cells, and can modulate both neural and immune systems [132]. The peptide signals through a G-protein-coupled receptor named calcitonin-like receptor that makes up the common core of receptors of the family (in particular CGRP and adrenomedullin) [133]. To dictate receptor specificity (and binding capabilities), calcitonin-like receptor requires accessory proteins for its function, the single-transmembrane accessory proteins named receptor activity modifying proteins (RAMP) 1–3. In particular, CGRP receptor is composed of the association of calcitonin-like receptor with RAMP1 [134]. This and the other receptor complexes of the family interact with an additional cytoplasmic protein, the CGRP receptor component protein (RCP). RCP is the functional link with the intracellular signalling pathways and, in particular, with cAMP formation [135, 136]. Activation of the CGRP receptor results in G*α*s-mediated increase in cAMP and subsequent activation of protein kinase A [137]. CGRP receptor, however, can couple to additional G proteins (as well as to other proteins, such as arrestins) to confer signalling. This includes G*α*q/11-mediated phospholipase C-*β*1 activation, intracellular calcium increase, mitogen-activated protein kinase (MAPK) cascade activation, and NO production [138–141]. Cellular background and context-dependence play a critical role as determinants for the activation of specific signalling pathways, as shown, for example, for calcium and MAPK [139, 141, 142].

As suggested for other neuropeptides, CGRP is thought to maintain homeostasis [143–145], a role which seems to be evolutionarily conserved as it has been assigned also to calcitonin (a peptide of the family) in zebrafish [146]. Coherently with a homeostatic role, CGRP can promote neurogenic inflammation in the periphery [147] and brain during headache [148] but also protect against local (LPS-induced) inflammatory or immune-mediated injuries [149, 150]. The peptide can directly induce the secretion of a variety of cytokines from T cells [151] and regulate the functions of several immune cells, including macrophages, T and B lymphocytes, and Langerhans cells [152–156].

In the brain, its receptors are expressed in all neural cells [81].* In vitro*, CGRP potently inhibits the secretion of proinflammatory mediators from LPS-activated microglia in a dose-dependent manner [157]. This effect is carried out also by adrenomedullin, a closely related peptide of the family, and spreads to involve astrocytes, when cocultured with microglia [157].* In vivo*, CGRP has been applied intrathecally (in the lumbar cerebrospinal fluid) during the induction phase of the experimental autoimmune encephalopathy, an animal model of multiple sclerosis. In this model and in multiple sclerosis, inflammatory microglia activation is thought to be involved from the early stages of the pathogenesis [158–161]. CGRP delivery into the cerebrospinal fluid reduced chronic experimental autoimmune encephalopathy signs while inhibiting microglia activation [81]. The analysis of RCP (probe for CGRP receptor involvement) showed that, in a relapsing-remitting form of experimental autoimmune encephalopathy, its expression increased during the relapsing phase in parallel with an increase in its nuclear localization in microglia. The nuclear localization of RCP could be induced also by CGRP itself both in cultured cells* in vitro* and in microglia* in vivo* following the peptide delivery in the cerebrospinal fluid during experimental autoimmune encephalopathy [81].

CGRP can regulate several pathways. For example, it was shown to interfere with TLR signalling, as seen in murine dendritic cells in which the peptide potently inhibited TLR-stimulated production of inflammatory mediators [162]. In addition the peptide exerts an antioxidant activity, as described* in vitro* [163–166], in an isolated heart preparation [167], and* in vivo* [168, 169].

## 9. Apelin-13

Apelin was identified as the endogenous ligand of the G protein-coupled receptor APJ, and its name derives from this discovery (i.e., APJ endogenous ligand) [170]. Following the purification from stomach extracts, a 36-amino acid peptide was isolated and sequenced (apelin-36). The corresponding cDNA identified a preproprotein of 77 amino acids containing the 36-amino acid peptide, apelin (42–77). Sequence alignment from different species revealed a strong conservation of the entire amino acid sequence, with a highest identity in the C-terminal region [171]. However, the potential existence of additional ligands was also hypothesized on the basis of the presence of two conserved basic doublets, apelin-17 and apelin-13 [172].

The receptor was thought to be involved in HIV infection, and so its ligand. However, the range of its functions widened soon [173]. At present, apelin is considered to be a pleiotropic peptide with a homeostatic function (as other neuropeptides). Its receptor, APJ, is expressed in the CNS at high levels, its mRNA expression being intense in the hypothalamic paraventricular and supraoptic nuclei and in the anterior and intermediate lobe of the pituitary, the pineal gland and nuclei of the olfactory system [174, 175]. Outside the brain, expression of the amphibian apelin receptor was first associated with an endothelial lineage [176] and it was later shown to be expressed in human heart and saphenous vein [177]; it can be upregulated in peripheral blood mononuclear cells [178]. Its actions include roles in the cardiovascular system [179], ischemia/reperfusion injury [180], pulmonary arterial hypertension [181], feeding behavior, gastrointestinal function, and fluid homeostasis [182].

The intracellular cascades activated by apelins include inhibition of production of cAMP (in CHO cells stably transfected with the apelin receptor [174]) and rise in intracellular calcium concentrations [183], and activate ERKs via a PTX-sensitive G protein and a PKC isoform in CHO cells expressing the murine apelin receptor [184].

Apelin-13 was found to exert a neuroprotective action in a stroke model. Following a transient focal ischemia/reperfusion injury model, the intracerebroventricular administration of the peptide at the onset of reperfusion reduced the infarct volume and the neurological deficits and inhibited the increase of myeloperoxidase activity. Moreover, the peptide was shown to decrease the expression of inflammatory cytokines as well as that one of Iba1 and GFAP indicating inhibition of microglia and astrocytes [185]. It is unclear whether apelin-13 can target directly microglia in this stroke model, and it is worth noting that its receptor was not detected neither in cultured microglial cells [183] nor in spinal cord microglial cells of SOD1(G93A) mice, a model of amyotrophic lateral sclerosis. Interestingly, however, apelin-13 and two structural analogues showed inhibition of mitochondrial ROS in cardiac cells, and, in a model of ischemia/reperfusion injury, preischemic infusion significantly reduced ROS formation and attenuated cell membrane damage [186].

## 10. Conclusion

There is a general agreement that microglia are a main homeostatic player in the brain, during development, by influencing synaptic activity and circuitry, and in pathological conditions. Although potentially beneficial, microglia activation can lead to a priming effect that can produce deleterious (instead of protective), self-sustaining effects. There is a growing body of evidence that a derangement from its physiological function transforms microglia in a major detrimental player in a variety of brain pathological conditions, ranging from trauma, infection, neuroinflammation, and neurodegeneration. This maladaptive microglial response needs to be targeted to dampen damage, invert vicious cycles, and restore cellular functions.

Among the modulators of microglia activation, neuropeptides have been recently used* in vivo* in different brain disease models. In addition to a potent* in vitro* effect, VIP, PACAP, CGRP, and apelin-13 have been shown to be able to dampen microglia activation also* in vivo* and to produce strong beneficial effects or at least reduce the symptom severity. Most of the administration procedures have used a delivery timing which cannot allow a clear-cut indication for a therapeutical effect of these molecules at present, but the very recent results of PACAP application in stroke are, instead, in this line [129]. Even though the pleiotropic (and homeostatic) effects of the neuropeptides imply that their action have to be analyzed carefully in each specific pathological condition (see, e.g., the neuroprotective effect of a substance P antagonist in a Parkinson's model [187]), it can be suggested that the neuropeptides are well suited to act as inhibitors of microglia activation in different brain disease models* in vivo*. Their microglial modulatory effects are accompanied by significant symptom amelioration and it can thus be anticipated that they may well become part of the therapeutical repertoire for brain diseases in the future.

## Figures and Tables

**Table 1:** *In vivo* administration of neuropeptides in the central nervous system produces beneficial effects in experimental models of brain diseases.

Neuropeptide	Experimental model	Delivery method/place	Administration: single/chronic	Administration timing	Effects on pathology (1), microglia (2) [timing of assessment]	Ref.
VIP	Brain trauma: vertical stab wound operation in periventricular area by glass micropipette	Micropipette/the same place as trauma	Single	The same time as trauma	(1) Reduced neuronal cell loss and inflammatory cytokine expression, (2) reduced microglia activation (CD11b/Mac1 expression) [5 days]	[108]

VIP	MPTP toxicity (Parkinson's disease model)	Micropipette? (not described)/substantia nigra	Single	One hour after i.p. injection of MPTP	(1) Reduced nigro-striatal dopaminergic neuron loss [7 days] and inflammatory cytokine/iNOS expression [12 hrs], (2) reduced microglia activation (CD11b/Mac1 expression) [24 hrs]	[109]

PACAP (+hMSCs)	Spinal cord injury (by weight drop device, 127)	Microsyringe (31-gauge needle)/spinal cord	Single	7 days after injury (the same time for PACAP and hMSCs)	(1) Increased motor functional recovery [31 days], (2) increased sparing of fibers [31 days], (3) increased level of antioxidant enzymes [7? 14? days]	[128]

PACAP	Permanent focal ischemia	Transplanted PACAP-producing stem cells/i.c.v.	Chronic PACAP production	Transplant: 3 days after ischemia	(1) Decreased neurological severity score [7, 14 days], (2) increased motor coordination [7, 14 days], (3) increased anti-inflammatory and decreased proinflammatory cytokines [7 days], (4) microglia: decreased cell number, increased ramification, Arg-1 and Ym-1 expression [7 days]	[129]

CGRP	EAE (MS model)	Osmotic minipumps/intrathecal (lumbar CSF)	Chronic	2 days after immunization	(1) Decreased motor impairment score, (2) microglia: decreased morphological activation, increased nuclear localization of a receptor component protein [15 days]	[81]

Apelin-13	Transient focal ischemia/reperfusion (stroke model)	i.c.v.	Single	The same time as reperfusion	(1) Decreased neurological score, reduction of infarct volume, decreased myeloperoxidase activity, decreased inflammatory markers, (2) decreased microglia cell number [24 hrs]	[185]

MPTP: 1-methyl-4-phenyl-1, 2, 3, 6-tetrahydropyridine; hMSCs: human mesenchymal stem cells; i.c.v.: intracerebroventricularly; EAE: experimental autoimmune encephalomyelitis; MS: multiple sclerosis; CSF: cerebrospinal fluid.
